# Vitamin C and the Lens: New Insights into Delaying the Onset of Cataract

**DOI:** 10.3390/nu12103142

**Published:** 2020-10-14

**Authors:** Julie C Lim, Mariana Caballero Arredondo, Andrea J. Braakhuis, Paul J. Donaldson

**Affiliations:** 1 Department of Physiology, New Zealand National Eye Centre, Faculty of Medical and Health Sciences, The University of Auckland, Auckland 1142, New Zealand; p.donaldson@auckland.ac.nz; 2 Discipline of Nutrition, Faculty of Medical and Health Sciences, The University of Auckland, Auckland 1142, New Zealand; mariana.cbllro@gmail.com (M.C.A.); a.braakhuis@auckland.ac.nz (A.J.B.)

**Keywords:** vitamin C, lens, cataract, oxidative stress, vitreous humor, vitrectomy

## Abstract

Cataracts or clouding of the lens is the leading cause of blindness in the world. Age and diabetes are major risk factors, and with an increasing aging and diabetic population, the burden of cataracts will grow. Cataract surgery is an effective way to restore vision; however, alternatives to cataract surgery are required to reduce the looming cataract epidemic. Since it is well established that oxidative damage plays a major role in the etiology of cataracts, antioxidants have been promoted as therapies to delay and/or prevent cataracts. However, many antioxidant interventions including vitamin C have produced mixed results as anti-cataract therapies. Progress has been made towards our understanding of lens physiology and the mechanisms involved in the delivery and uptake of antioxidants to the lens which may guide future studies aimed at addressing some of the inconsistencies seen in previous animal and human studies. Of interest is the potential for vitamin C based supplements in delaying the onset of cataracts post vitrectomy which occurs in up to 80% of patients within two years. These targeted approaches are required to reduce the burden of cataract on hospitals and improve the quality of life of our aging and diabetic population.

## 1. Introduction

With an aging and diabetic population, the number of individuals with major eye diseases is increasing, and vision loss in the elderly is projected to be a major public health problem. Cataract or the clouding of the lens is the leading cause of blindness and is responsible for 51% of global blindness [1]. Age is a major risk factor for cataracts [2,3], with the disease progressing gradually, appearing first in the fourth or fifth decade, but not affecting vision until typically the sixth decade. Diabetes is another risk factor, with diabetic patients 2–5 times more at risk for developing cataracts and at an earlier age [4]. The only available treatment for cataract is surgery. This involves replacement of the cataractous lens with an artificial plastic lens which effectively restores sight. However, insufficient surgical facilities in poor and developing countries, and long waiting lists in developed countries, means that alternatives to cataract surgery are required. It has been calculated that delaying the onset of cataract by 10 years would halve its incidence, and therefore reduce the need for, and cost associated with, cataract surgery [5]. Because of the proven association between lens cataract and oxidative damage, antioxidant supplementation has been promoted as a treatment strategy to slow the progression of cataract [6,7,8]. However, antioxidant supplementation has proven to be largely ineffective as an anti-cataract therapy.

Vitamin C (also known as L-ascorbate or L-ascorbic acid) is present in the lens and surrounding ocular humors, which bathe the lens at a concentration 50-fold higher than that found in plasma [9,10]. It acts as a physiological “sunscreen” to protect the lens from UV (ultraviolet light) induced oxidative damage, and to regenerate vitamin E and glutathione to further increase antioxidant capacity. With advancing age, vitamin C levels in the lens decrease and a decrease in vitamin C in the lens is associated with increasing cataract severity [11]. Consumption of additional dietary vitamin C can increase the concentration of vitamin C in the lens [12], and there is evidence that the incidence of cataract may be higher in persons who have a low plasma concentration of vitamin C [12]. This indicates that vitamin C supplementation may help to replenish and restore vitamin C levels as we age to protect against cataract.

The purpose of this review is to consolidate animal and more recent epidemiological studies to determine future areas of research that could provide more clarity about the role of vitamin C in the lens. By combining our current understanding of lens structure and physiology and the delivery and uptake of antioxidants and nutrients to the different region of the lens [13,14], this review provides new areas of research which can be used to re-evaluate and re-design nutrition based studies. The latter should help provide a more clear and consistent view on whether vitamin C supplementation is beneficial to the lens and whether it affords protective against specific types of cataract. Of particular interest is the potential of vitamin C supplementation to prevent cataract following vitrectomy surgery. Vitrectomy-patients have a high chance of developing cataracts within two years post-vitrectomy [15], providing a unique window with which to test nutritional strategies without many of the variables encountered when studying populations over long periods of time. Hence, an enhanced knowledge on vitamin C pathways in the eye will be key to the design of targeted nutritional strategies aimed at reducing the onset of cataract to avoid the looming cataract epidemic.

## 2. The Cataract Epidemic

Cataracts are the leading cause of blindness accounting for 51% of global blindness [1]. Given our globally aging population, the social and economic costs of cataract are quite staggering and the demand for cataract surgery far exceeds limited public health resources. In 2010, there were 10.8 million cataract blind people [16], with this number expected to increase to 40 million in 2025 as the population grows and ages, with greater life expectancies [17]. In many countries, cataract surgery remains one of the most commonly performed procedures, with ~8 million cataract operations performed each year worldwide with an additional ~10 million people added to a backlogged system because of the lack of appropriate cataract surgery services in the areas of need [18]. Although the majority of cataracts are due to the aging process [3,19], children can be born with the condition as a result of an inherited genetic condition, or a cataract may develop as a result of a medical condition such as diabetes, other eye diseases, injuries [20], or past eye surgery such as vitrectomy [21].

## 3. Aetiology of the Different Types of Cataract

**Figure 1:**
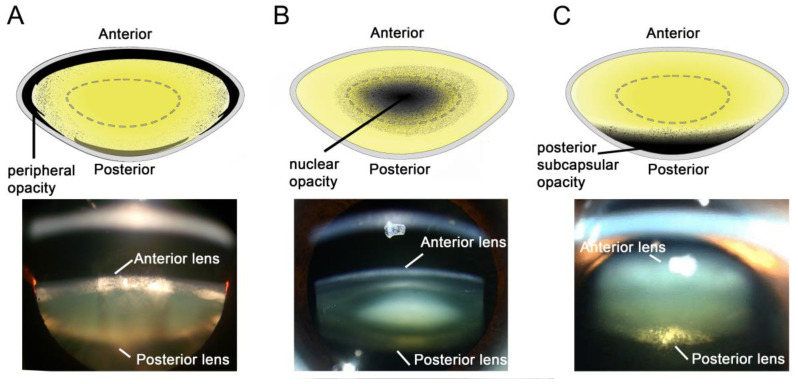
Location of cataract subtypes. Schematic diagrams and scheimpflug slit-lamp photographic images showing the three main types of cataract: (**A**) cortical, (**B**) nuclear, and (**C**) posterior subcapsular (PSC). Source: (**A**) From Uspal NG, Schapiro ES (February 2011). Cataracts as the initial manifestation of type 1 diabetes mellitus. Pediatric Emergency Care. 27 (2): 132–4. Attribution-ShareAlike 4.0 International (CC BY-SA 4.0). (**B**) Ophthalmic Atlas Images by EyeRounds.org, The University of Iowa licensed under a Creative Commons Attribution-NonCommercial-NoDerivatives 3.0 Unported License. (**C**) From Chaudhary M, Shah DN, Chaudhary, RP. Scleritis and Takayasu’s disease. Nepal J Ophthalmol 2017; Vol 9 (18): 170–174. Attribution-NonCommercial-NoDerivatives 4.0 International (CC BY-NC-ND 4.0).

Cataracts can form in different parts of the lens with three main type of cataracts classified according to the location in which the cataract first forms; cortical cataract which manifests as an opacity in the peripheral edges of the lens, and is highest amongst diabetic patients (Figure 1A) [22,23], nuclear cataract where the cataract first occurs in the nucleus, or centre of the lens, and is typically associated with aging (Figure 1B) [24], and posterior subcapsular cataract, which forms in the back of the lens, and is often associated with the use of certain medications, including corticosteroids and diabetes medications (Figure 1C) [25,26]. In addition, patients can present with opacity in more than one area of the lens which can cause overlap in the classification of cataracts.

### 3.1. Diabetic Cortical Cataracts

**Figure 2:**
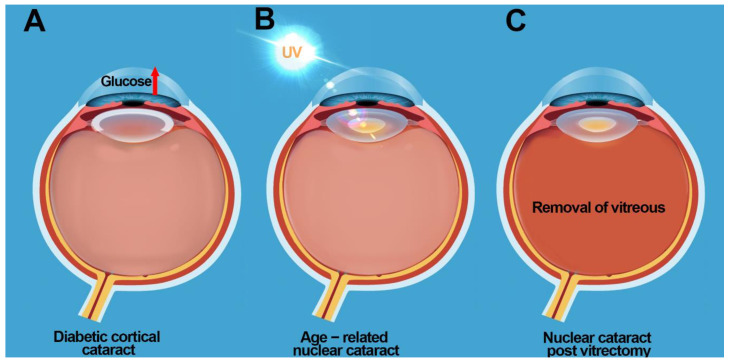
Schematic of the eye showing the development of (**A**) diabetic cortical cataract due to elevated levels of glucose, (**B**) age-related nuclear cataract due to UV exposure, and (**C**) nuclear cataract post vitrectomy due to depletion of vitamin C and elevated PO_2_ levels.

Diabetes leads to various complications including cataracts (Figure 2A) and with an increasing global prevalence of diabetes, the incidence of cataract formation is rising. Diabetic patients are more likely to get cataracts at an earlier age [27] with cataracts progressing faster in diabetics compared to non-diabetics [28]. The pathogenesis of diabetic cataract is attributed to the accumulation of the impermeable osmolyte, sorbitol, produced from excess glucose by the enzyme aldose reductase (AR), initiating osmotic stress [29,30]. This results in fluid accumulation, lens fibre cell swelling, and tissue liquefaction [29,30]. More recent evidence suggests that hyperglycaemia results in increased polyol activity which generates osmotic and oxidative stress in the diabetic lens [31]. This offers an explanation for the slow development of cataracts that is typically seen in the majority of adult diabetic patients [32]. While initially hyperglycaemia results in osmotic stress, the lens is able to regulate its volume through osmoregulatory mechanism that can accommodate small changes in osmotic pressure [33]. Over time however, the ability of the lens to actively regulate its volume becomes impaired [32] due to oxidative damage to the pathways that regulate fibre cell volume resulting in the localised zone of tissue liquefaction observed in diabetic cortical cataract. However, it should be noted that the association between aldose reductase, osmotic stress, oxidative stress, while very strong in the rat because of the high aldose reductase levels, is not supported by the various clinical trials with aldose reductase inhibitors. This is because while rat lenses have high levels of aldose reductase activity and low levels of sorbitol dehydrogenase activity [34,35], human lenses exhibit low aldose reductase activity and high sorbitol dehydrogenase activity [34]. As a result, using appropriate animal models of diabetic cataract that are translatable to human lenses will be important in identifying additional pathways that contribute to cataract formation [36].

### 3.2. Nuclear Cataracts

The most common form of cataract is age related nuclear cataract (Figure 2B) and is responsible for 50% to 90% of cataracts in developing countries [1,3]. The pathogenesis of age-related nuclear cataract is largely attributed to the chronic exposure of the lens to molecular oxygen, resulting in oxidative damage to proteins in the lens nucleus, protein aggregation, light scattering, and ultimately loss of lens transparency [37,38,39,40,41,42]. Under normal physiological conditions, the lens exists in a relatively low oxygen environment, with a partial pressure of oxygen <10 mm Hg around the lens [9,21,43]. Low oxygen environment together with high concentrations of vitamin C in the aqueous and vitreous humors [10,44] and high levels of glutathione (GSH) [45] and vitamin C [10] in the lens, ensures protection of the lens against oxidative stress. While vitamin C levels are known to decrease with age in the lens [11], it is unknown in which region of the lens vitamin C depletion initially occurs. However, with increasing age, GSH is known to decrease specifically in the lens nucleus [46], rendering proteins in this region susceptible to oxidative damage. Because of the proven association between lens cataract and oxidative damage, antioxidant supplementation has been promoted as a treatment strategy to slow down the progression of this type of cataract [6,7,8].

Nuclear cataracts also occur as a secondary consequence of previous ocular surgery such as vitrectomy (Figure 2C). Vitrectomy is a procedure in which the vitreous humor at the back of the eye is removed. Vitrectomy procedures are often done to allow surgeons access to the back of the eye, during operations for retinal conditions, or to drain vitreous fluid filled with blood (common in a person with diabetes), floaters, or clumps of tissue that would obscure vision. While vitrectomy may help to repair damaged or scarred retina or clear the vitreous of debris, studies report that vitrectomy causes rapid progression of nuclear cataracts resulting in the need for cataract surgery in 60–95% of patients within two years [47,48,49,50,51]. As a result, patients who have had to endure the anxiety and stress associated with vitrectomy, are now faced with the prospect of additional surgeries for treatment of cataract.

The molecular mechanisms of cataract formation post vitrectomy was elucidated by David Beebe and colleagues who showed that this was linked to depletion of vitamin C in the vitreous and loss of the tightly managed oxygen gradient [9,52,53,54]. Under physiological conditions, oxygen enters the eye by diffusion from the retinal vasculature and through the cornea. The lens consumes oxygen to maintain its hypoxic state, while simultaneously, the vitreous humor consumes oxygen through vitamin C. In the vitreous chamber between the retina and the lens there is a decreasing gradient of oxygen, with the partial pressure of oxygen (PO_2_) ranging from 22 mmHg close to the retina and ~9 mmHg close to the lens [55]. However, vitrectomy disrupts this oxygen gradient, and without the constraints of a gel-like vitreous humour, oxygen is able to freely mix though the vitreous chamber resulting in consumption of vitamin C and elevation of oxygen tension levels to ~14 mmHg near the lens [9,55,56]. These abnormally high levels of oxygen persist over many months after the initial surgery [21] and over time lead to elevated oxidative stress in the lens and the formation of nuclear cataracts.

In all three types of cataract described above, it is clear that oxidative stress plays a major role in cataract formation. Vitamin C plays a critical role in consuming oxygen and maintaining low levels of oxygen within the eye, suggesting that replenishing vitamin C in the lens and vitreous is a viable strategy for minimizing oxidative stress and reducing the risk of cataract formation. In the next section, we will describe the roles and biochemical properties of vitamin C in the lens before reviewing a selection of animal studies and human intervention studies investigating the ability of vitamin C supplementation to reduce the risk of cataract.

## 4. Roles of Vitamin C in the Eye

In humans, high concentrations of vitamin C exist in the aqueous and vitreous humor exceeding plasma concentrations by as much as 20- to 70-fold [9]. Interestingly, vitamin C levels in the ocular humors are quite different between nocturnal and diurnal animals with vitamin C levels much higher in the ocular humors of humans compared to rats [57]. This has led to the suggestion that vitamin C may play a protective role in those animals who are most exposed to light [57]. In humans, the high concentrations of vitamin C in the aqueous humor, together with its ability to absorb UV light, have led to its referral as a physiological “sunscreen” [58], preventing the penetration of UV light and photo-induced oxidative damage to tissues. Vitamin C is effective in scavenging or quenching the superoxide radical anion, hydrogen peroxide, hydroxyl radical, singlet oxygen, and reactive nitrogen oxide [59], with several studies reporting that vitamin C in the aqueous humor acts to protect the cornea, lens, and other ocular tissues against oxidative damage [60,61,62,63]. Vitamin C also protects the reducing powers of other antioxidants such as *α*-tocopherol (vitamin E) by rescuing *α*-tocopheryl radicals in membranes [64]. In the lens, vitamin C has been shown to play a role in prevention of membrane lipid peroxidation [65] and in protection against light induced oxidative damage to the Na^+^K^+^-ATPase pump [63].

## 5. Biochemical Properties of Vitamin C

**Figure 3:**
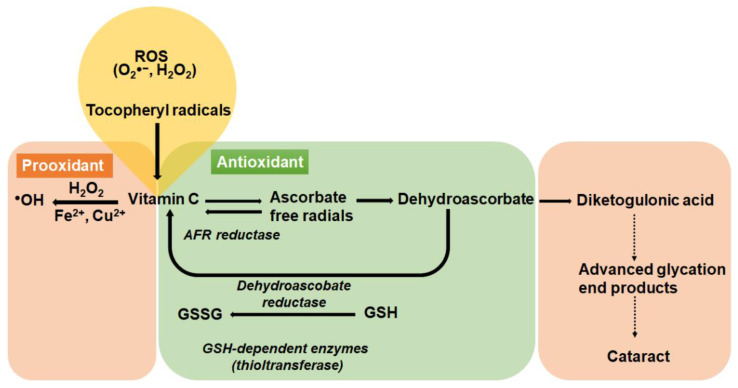
The redox pair vitamin C/DHA. The antioxidant vitamin C is oxidised first to the ascorbate radical and then to DHA which are both reversible reactions due to the enzymes AFR reductase and DHA reductase which relies on GSH as a co-factor. With continued oxidative stress, DHA undergoes an irreversible rearrangement to diketogulonic acid which is linked to accelerated protein cross linking and cataract formation. Vitamin C can also act as a pro-oxidant by reducing metal ions that generate free radicals through the Fenton reaction.

Vitamin C’s antioxidant properties are due to its ability to donate electrons to free radicals from both the second and third carbon and quench their reactivity [66]. Most animals are able to synthesize vitamin C endogenously. The exceptions are humans, guinea pigs, some fish, birds, and insects [67]. In humans, the conversion of l-gulono-γ-lactone into vitamin C, which is catalyzed by the enzyme gulonolactone oxidase is not functional, due to the accumulation of several mutations that has turned the gene into a non-functional pseudogene [68], meaning that humans must rely on dietary intake of vitamin C. In the process of detoxifying reactive oxygen species, vitamin C becomes oxidized to dehydroascorbate (DHA). However, DHA can be reduced back to vitamin C to regenerate vitamin C pools either via glutathione-dependent enzymes or nonenzymatically using low molecular weight antioxidants such as glutathione or cysteine (Figure 3). In the presence of continued oxidative stress, DHA undergoes irreversible degradation to diketogulonic acid which is implicated in the modification and crosslinking of lens proteins [69] (Figure 3). Under pathological conditions or at high doses, vitamin C in the presence of redox-active ions such as iron or copper, can act as a pro-oxidant contributing to the formation of hydroxy radicals via the Fenton reaction (Figure 3) that can lead to significant oxidative damage [70]. This means that vitamin C can switch from being an antioxidant under physiological conditions to a pro-oxidant under pathological conditions.

## 6. Transport of Vitamin C into the Ocular Humors

Early in development, the embryonic human lens is nourished by an external blood supply known as the tunica vasculosa lentis, which is transient, and regresses during the course of development, so that by the fetal period the lens is avascular [71]. While this loss of vasculature is essential for ensuring that light is not absorbed by haem pigments [72], it means that the lens is reliant on the aqueous humor for its nutrients and antioxidants [73].

The aqueous humor is continuously formed from plasma (~2.5 μL/min in humans) and is secreted by the ciliary epithelium. This double layer of epithelium is composed of a pigmented epithelium (PE), which interfaces with the highly vascularized stromal tissue that contains fenestrated capillaries, and a non-pigmented epithelium (NPE) which interfaces with the aqueous humor [73]. The PE and NPE are joined at their apical membranes by gap junctions which forms a functional subunit for aqueous humor section. Aqueous humor formation is driven by chloride (Cl^−^) secretion mediated by the PE-NPE pair and involves stromal Cl^−^ entry into PE cells, diffusion through gap junctions and NPE cell secretion of Cl^−^ into the anterior chamber of the eye [73].

**Figure 4:**
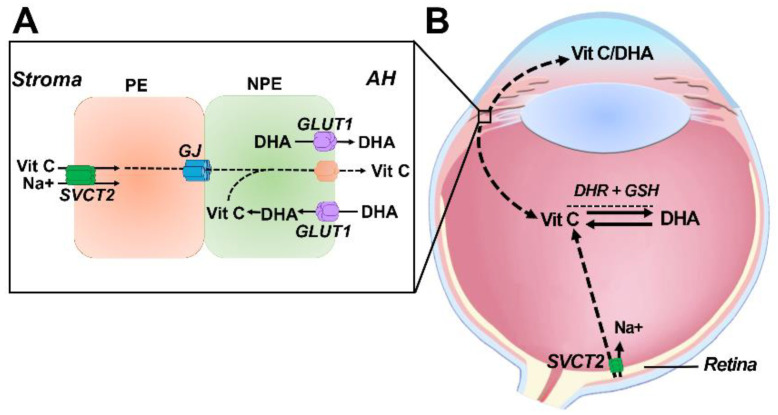
Molecular mechanisms involved in the secretion of vitamin C and DHA by the ciliary epithelium into the ocular humors. (**A**). Located in the apical surface of the PE cell which interfaces with the stroma microvasculature is SVCT2 which transports vitamin C resulting in the high accumulation of vitamin C in the PE. Vitamin C then moves via passive diffusion though a gap junction medicated pathway into the NPE where it then is transported out of the NPE by an unidentified mechanism into the aqueous humor (AH). Depending on the concentration gradient for DHA, GLUT1 which is located in the apical surface of the NPE cells, can either be used to transport DHA from the NPE into the AH, or DHA from the AH can be taken up into the NPE and recycled back to vitamin C. (**B**) Vitamin C is secreted into the aqueous humor and the vitreous humor. Transport of vitamin C via SVCT2 expressed in retinal pigment epithelial cells and active regeneration of vitamin C from DHA are complementary mechanisms most likely used to sustain high levels of vitamin C in the vitreous. PE-pigmented epithelium; NPE-non pigmented epithelium.

In the human ciliary epithelium, vitamin C uptake from the stroma is mediated by the Na^+^ dependent vitamin C transporter, SVCT2 [74] which was shown to be expressed in the PE [13]. From the PE layer, vitamin C is proposed to diffuse via gap junctions to the NPE (Figure 4A). However, it is unknown how vitamin C in the NPE is transported into the anterior chamber, suggesting unidentified active transporters must be involved. DHA can also be secreted by the NPE cells given that the facilitative glucose transporter GLUT1, the major transporter of DHA (and glucose), is expressed in the NPE layer [13]. However, it has been suggested that the concentration gradient for DHA would most likely suggest that the function of GLUT1 in NPE cells is for the local recycling of DHA back to vitamin C in which DHA is taken up from the anterior chamber into the ciliary epithelium where it is regenerated to vitamin C and then secreted back into the aqueous. In other animals, it should be noted that vitamin C transporter expression is different [13]. In the mouse eye, there is an absence of SVCT2 in the ciliary epithelium, but high expression of SVCT2 in the retina suggesting that the retina is the more likely source of vitamin C in the vitreous for nocturnal animals [13]. This may explain the lower levels of vitamin C in the aqueous humor in mice compared with diurnal species such as humans.

In humans, vitamin C levels are even higher in the vitreous humor compared to the aqueous humor. While the ciliary body is likely to be a source of vitamin C in the vitreous, other mechanisms must be required to maintain and sustain high levels of vitamin C in the vitreous. The strong expression of SVCT2 in the human retinal pigmented epithelium and other layers of the retina [13] may indicate that in addition to the ciliary epithelium the retina may serve as a source for vitamin C in the vitreous gel (Figure 4B). Recently, human donor lenses have been shown to export GSH from its posterior surface suggesting that this source of GSH could be used to recycle DHA back to vitamin C [75]

## 7. Delivery and Uptake of Vitamin C and DHA into the Lens

**Figure 5:**
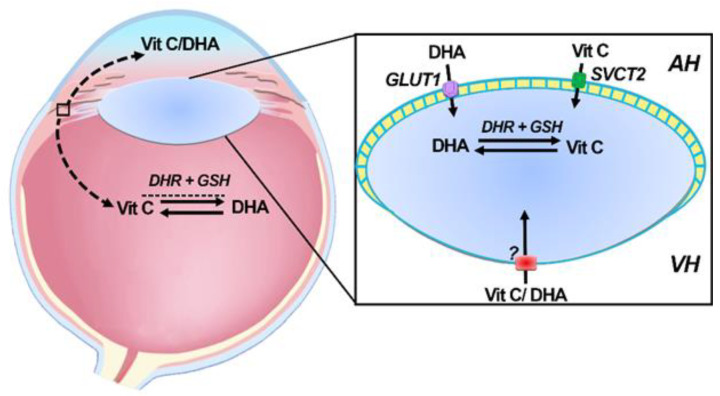
Vitamin C uptake pathways in the lens. In human lenses, SVCT2 localized to the lens epithelium is used to accumulate vitamin C from the aqueous humor (AH), while GLUT1 expressed in the epithelium and cortical fiber cells can function to uptake DHA from the aqueous humor. Whether vitamin C and/or DHA in the vitreous humor (VH) can be taken up from the posterior side of the lens is unknown.

Uptake of vitamin C in the lens occurs by transport of both vitamin C and DHA (Figure 5). In human epithelial cells, vitamin C uptake was Na^+^-dependent with molecular analysis revealing SVCT2 to be the likely transporter involved [76]. SVCT2 gene expression is upregulated in response to oxidants suggesting that vitamin C uptake can increase under oxidative stress conditions [76]. The water channel AQP0 may also be involved in the permeation of vitamin C into lens cortical fiber cells [77] since its expression was increased in diabetic rats and upon vitamin C treatment [78].

Although DHA in the aqueous humor constitutes only about 10% of total vitamin C content, it is DHA which appears to be preferentially transported into the lens and then recycled back to vitamin C [79]. This accumulation is mediated by facilitative glucose transporters of which GLUT1 has been shown to expressed in the epithelium and fiber cells of human donor lenses [80]. While the lens is presumably able to source vitamin C and/or DHA from vitreous humor (Figure 5), it is unknown whether SVCT2 and GLUT1 are expressed at the posterior surface of the lens in order to facilitate this.

## 8. Evidence of the Effects of Vitamin C on Cataract Prevention

Given the protective effects of vitamin C in the lens and the link between a decrease in vitamin C with increasing age and with increasing cataract severity [11], it is not surprising that numerous studies exist investigating the relationship between vitamin C and the risk of cataract. While there a number of excellent in depth reviews on vitamin supplementation, diet, and cataract in human populations [7,8,81,82,83,84,85], reviews summarizing the findings from animal studies are lacking. However, these studies are important as they should be used to help inform and guide the design of human therapeutic studies.

### 8.1. Animal Studies

The evidence of vitamin C as an anticataract agent in animal studies has remained elusive and difficult to prove. In reviewing the literature, there did not appear to be a consistent approach towards studying the efficacy of vitamin C on the lens. For example, there were differences in species selection and the use of nocturnal versus diurnal animals (rodents versus guinea pigs), the method of cataract induction (UV exposure, selenite, buthionine sulfoximine), the type of cataract induced (nuclear vs. cortical cataract), and different set of parameters that were used to assess the ability of vitamin C to protect against cataract (see Table 1). In this section, we provide a selective summary of animal studies in order to demonstrate the type of studies that have been conducted on the lens, and to reflect on how we can develop a more consistent approach that utilizes a standard set of parameters to test the efficacy of vitamin C in appropriate animal models that best mimic the cataract process observed in humans.

#### 8.1.1. The Antioxidant Role of Vitamin C in the Lens

In vitro studies have demonstrated that vitamin C protects rodent lenses from oxidative damage induced by UV-B exposure [86], hydrogen peroxide [87], and other ROS-inducing agents [88]. In vivo studies have also reported a protective effect of vitamin C on the lens. Diurnal guinea pigs which have naturally higher vitamin C levels in the aqueous humor, and like humans rely on a diet supplemented with vitamin C, were shown to be more protected against UV-B induced DNA damage to the lens epithelium compared to vitamin C deficient guinea pigs [93]. Guinea pigs placed on high dietary vitamin C (50 mg/day) contained over three times more vitamin C in the lenses than guinea pigs fed low dietary vitamin C (2 mg/day) [94]. In addition, lenses from high dietary vitamin C fed animals contained less high-molecular-weight aggregates following UV exposure compared to low dietary vitamin C fed animals [94]. Knockout mice which cannot synthesize vitamin C due to genetic disruption of the gluconolactonase gene were fed a vitamin C sufficient diet (1.5 g/L) and then exposed to UV-B. This resulted in less extensive opacities compared to knockout mice fed a vitamin C deficient diet (0.0375 g/L) (Ishikawa et al., 2012) [96]. In a selenite model of cataract, treatment of rat pups with vitamin C exerted a marked protective effect against the development of nuclear cataracts compared to those pups that did not receive vitamin C [95]. Biochemical analysis of lenses revealed that selenite plus vitamin C treatment helped to maintain ATP and GSH levels, and resulted in reduced malondialdehyde (MDA) levels, a marker of lipid peroxidation. Streptozotocin (STZ)-induced diabetic rat models have also shown that dietary vitamin C supplementation is beneficial to the lens. For example, dietary vitamin C supplementation was shown to relieve oxidative stress in STZ-induced diabetic aged rats by minimizing peroxidation levels and enhancing glutathione peroxidase activity in the lens [97]. In another study, dietary vitamin C supplementation of STZ-induced diabetic rats resulted in a reduction in cataracts and a decrease of γ-crystallin leakage into the ocular humors [98]. Finally, intraperitoneally administered vitamin C, vitamin E or selenium, or a combination of Vitamin E and selenium in STZ induced diabetic rats revealed that while vitamin C, vitamin E, and selenium can all protect the lens against oxidative damage, the effect of vitamin C appeared to be much greater than that of vitamin E and selenium [99].

#### 8.1.2. The Pro-Oxidant Role of Vitamin C in the Lens

While the above studies demonstrate a protective effect on vitamin C on the lens, other studies suggest a role of vitamin C in stimulating the progression of cataracts. A recent study have revealed that vitamin C in the lens is a source of oxoaldehyde stress that can be beneficial by promoting chaperone activity, or detrimental by removing protein charges [102]. Vitamin C is also know to act as a pro-oxidant due to the metal catalysed reaction of vitamin C which produces ascorbate free radicals, DHA and H_2_O_2_ which are toxic to the lens and if not reduced by a mechanism such as the GSH redox cycle, can result in the formation of highly reactive carbonyls [103]. This results in rapid glycation of lens proteins [104] and the formation of protein crosslinks capable of scattering visible light [69]. In vitro cross linking of lens crystallin proteins occurs rapidly in the presence of vitamin C (20 mM) and air due to the oxidation products of vitamin C [90,92,105]. It has been suggested that vitamin C can make a larger contribution to cross-linking than glucose and that as a result vitamin C is a significant glycating agent [106]. In vivo studies have shown that guinea pigs supplemented with 5.5 mM vitamin C for four weeks in their drinking water and then exposed to ultraviolet-B (UV-B) radiation were not protected against UV-B induced cataract [101]. Overexpression of SVCT2 in mouse lenses, which typically have low levels of vitamin C and SVCT2 transporter activity, resulted in elevated levels of vitamin C and its associated oxidation products in the lens [100]. In addition, transgenic lenses exhibited a yellow colour and accelerated modification of crystallin proteins by the Maillard reaction [100]. These results are consistent with changes reported for human lenses during normal aging and cataract formation [107,108] suggesting that vitamin C oxidation plays a role in human lens aging and cataract.

Taken together, the available evidence suggests that while maintenance of vitamin C levels are required to prevent oxidative damage to the lens, excessive administration of vitamin C appears to be linked to cataract formation. However, it is difficult to assess from these animal studies the benefits versus risk value of higher than normal intake levels. However, since these animal studies were conducted, our knowledge on lens physiology has significantly grown. It is now accepted that the lens depends on an internal microcirculation system to deliver nutrients and antioxidants to the deeper regions of the lens [7,14]. This opens up new areas of research into vitamin C delivery and uptake into the different regions of the lens and investigations into whether this delivery can be enhanced to provide protection under conditions of oxidative stress. Looking ahead, it will also be important to consider the choice of animal model given that diurnal and nocturnal species exhibit marked differences in their baseline vitamin C levels, the expression of vitamin C uptake transporters, and the ability to synthesize vitamin C. In general it appears that in rodent models of cataract there are benefits of vitamin C supplementation in the prevention or delaying of opacities. However, like most other interventions in other rodent disease models, rodents respond well because the stress is acute and high drug levels can be easily achieved. Furthermore, all rodent strains are inbred, and thus the number of pathogenic pathways is limited. In addition, the selection of the cataract model is equally important as it needs to mimics changes typically associated with age related or diabetic cataract in humans The morphological, physiological and biochemical changes associated with age related and diabetic cataracts are different [36,109], and so the parameters used to assess the efficacy of vitamin C will also be different. Finally, a standard set of parameters or biomarkers should be used amongst researchers to provide more consistent measurement of the relationship between vitamin C and cataract progression that can be used to aid the translation of animal work into human studies

### 8.2. Evidence of the Effects of Supplemental or Dietary Vitamin C on the Prevention of Cataracts in Humans

Given the role of oxidative stress in cataractogenesis, it is not surprising that the role of antioxidant intake and cataract in human populations has been extensively studied [6,8,110]. While some studies generally support the association an increased intake of vitamin C and other antioxidant nutrients with a decreased risk of cataract [111,112], longer term clinical trials do not tend to support this conclusion, indicating that vitamin C had little or no benefit for treatment durations up to 6.5 years. The Linxian cataract study conducted in a nutritionally deficient population in China (2, 3249 participants aged 45 to 74 years) involved the random assignment of participants to a daily supplement of 14 vitamins and 12 minerals at 2 to 3 times the U.S. recommended dietary allowance. Compared to placebo, a vitamin C/mineral combination had no effect on reducing the prevalence of cataracts for treatment durations of up to seven years [113]. The Age-Related Eye Disease Study (AREDS) in which participants (4629 participants aged 55 to 80 years) were randomly assigned to receive daily oral tablets containing antioxidants (vitamin C, 500 mg; vitamin E, 400 IU; and beta carotene, 15 mg) or no antioxidants found that a high-dose formulation of vitamin C, vitamin E, and beta carotene in well-nourished older adult cohort had no effect on the risk of development or progression of any cataract type [114]. The Roche European American Cataract Trial (REACT) conducted in the U.K. and U.S. in which participants (445 participants over the age of 40 years) received a daily oral antioxidant mixture (beta-carotene 18 mg; vitamin C, 750 mg; and vitamin E, 600 mg) found modest benefits in the U.S. cohort but no significant benefit in reducing the risk of cataract progression in the U.K. cohort [115]. In a more recent search of studies within the last 10 years (outlined in Table 2), Christen et al. reported on the first randomized trial to test the individual effect of vitamin C supplementation on the prevention of cataract. In this study, daily supplementation of 500 mg of vitamin C in healthy US male physicians 50 years or older revealed no effect on risk of cataract or cataract extraction after eight years of treatment [116]. Concerning were findings from the Swedish Mammography Cohort of women aged 49–83 years, which showed that vitamin C supplementation for longer than 10 years was associated with a 25% increase in the risk of cataract extraction. Among women aged 60 years and older, supplementation with vitamin C was associated with a 38% increased risk of cataract extraction [117]. In a follow up study, the risk of age related cataract was investigated in Swedish men and revealed that the use of multiple supplements in combination with vitamin C was not associated with cataract risk, but that the use of high dose vitamin C may increase the risk of cataract [118]. The use of dietary supplements in the form of multivitamins or a specific vitamin is widespread ranging from 22% to 53% in studies conducted from USA, Canada, Korea, UK, Sweden, Germany, and France [119,120,121,122,123,124,125]. While these supplements are taken for a range of health reasons, based on findings from the above clinical trials, the long-term value of vitamin C supplementation in decreasing the risk of cataract progression is questionable, and at high doses may in fact exacerbate cataract progression.

However, the general consensus of studies evaluating a well-balanced diet rich in fruit and vegetables tends to suggest that intake via a healthy diet enriched with Vitamin C may be a more optimal approach towards slowing down the progression of age related cataracts [126,127]. A search of the literature within the last 10 years found studies which support the evidence for a healthy diet enriched in Vitamin C and the reduced risk of cataract (outlined in Table 3). In the India Study of Age-related Eye Disease (INDEYE), which examined the association between vitamin C and age related cataract in the Indian population, plasma vitamin C and dietary vitamin C was inversely associated with cataract with the authors highlighting that this strong association with vitamin C and cataract in a vitamin C-depleted population may in part, explain the high levels of cataract in India [128]. The European Eye Study (EUREYE) study investigated the relationship between cataract, fruit and vegetable intake, and dietary and blood levels of carotenoids plus vitamins C and E in a Spanish population. High daily intakes of fruit and vegetables and vitamins C and E were associated with a significantly decreased prevalence of cataract or cataract surgery with daily dietary vitamin C intakes above 107 mg inversely associated with reduced odds of cataract [129]. In a U.S. based study assessing the association between healthy diet scores and prevalence of nuclear cataract in women, having a high healthy eating index (HEI) score was the strongest modifiable predictor of low prevalence of nuclear cataracts. Women with higher HEI scores had higher vitamin C intakes than those with lower scores with a trend for a protective association of vitamin C intake from foods alone, but not from a combination of foods and supplements, suggesting that vitamin C–containing foods rather than vitamin C itself may afford protection from nuclear cataract [130]. Theodoropoulou and colleagues conducted a case-control study to assess the association between diet and the risk of cataract in Greece. The results showed a protective association between cataract risk and intake of vitamins C and E and carotene, with an increase of 185 mg of vitamin C intake/day to reduce, at least by half, the risk of cataract overall, as well as nuclear and posterior subcapsular cataract [131].

While it appears that a diet high in fruit and vegetables containing vitamin C may be protective against cataracts, the longitudinal nature of nutritional studies and the number of uncontrolled variables present in populations over long periods of observations may affect the observed rates of cataract progression.

## 9. Cataract Prevention Post Vitrectomy: Restoring Antioxidant Balance in the Eye?

Cataract formation following vitrectomy is a well-recognized postoperative complication of the procedure with the incidence of cataract development as high as 80% within two years after surgery [47,48,49,50,51]. From a nutritional point of view, studying a cohort of individuals in which cataract develops within a two year time frame provides a much-shorted interval of observation than in studying age related cataract progression in the general population. This would minimize a participants potential exposure to uncontrolled variables and potentially allow the researcher to more definitely evaluate the efficacy of an intervention agent to delay the progression of cataracts.

Studies found through clinicaltrials.gov a database of privately and publicly funded international clinical studies identified two trials linked to testing interventions specifically aimed at delaying the progression of cataracts post vitrectomy. The first was a randomised double blind human clinical trial testing the efficacy administration of two doses of OT-551 eye drops in 164 patients (50 years and above) following vitrectomy (NCT00333060). It is unclear whether OT-551 was an antioxidant compound or whether the trial went ahead, but no outcomes or publications were reported from this trial. The second trial was also a randomised double blind human clinical trial testing the efficacy of Lenstatin^TM^, an over the counter oral antioxidant nutritional supplement to inhibit cataract post vitrectomy (NCT02131194). The formulation included Riboflavin, L-glutathione, C-phycocyanin, lipoic acid, pryruvate, alpha lipoic acid, quercitin, tumeric, silybin, lutein, zeaxanthin, and astaxanthin. Participants took two Lenstatin^TM^ capsules day versus placebo for six months post vitrectomy with lens densitometry measurements taken at baseline and at six month post-operatively. The study was underpowered in sample size with no significant difference in lens nuclear density between Lenstatin^TM^ and placebo groups [133].

With very few studies reported, future work re-examining the efficacy of vitamin C supplementation via the diet or through nutritional supplements will be of great interest in the future and may represent a cost effective solution in reducing the number of individuals requiring cataract surgery following vitrectomy.

## 10. Conclusions

In general, clinical trials by and large have failed to show convincing beneficial effects of Vitamin C supplementation on cataract incidence, except in those cases in which patients may have had low vitamin C levels to begin with. Unfortunately, only few studies included plasma vitamin C levels. Certainly there is no basis for supplementing with high doses since Vitamin C in excess of 250 mg/day is excreted in the urine. While it seems clear that a healthy diet rich in fruits and vegetables, coupled with healthy lifestyles can help reduce the risk factors for age related cataract, it is estimated that over 2 billion people do not have regular access to safe, nutritious, and sufficient food, and so further work is still required to find alternatives to delay the cataract epidemic caused by our increasing aging and diabetic population. Avoidance of risk factors such as diabetes, UV sunlight, and steroids should all be considered as part of a strategy to delay the progression of age-related and diabetic cataract. However integrating our knowledge of how the lens delivers and accumulates vitamin C and testing this in well-designed studies will play an important part towards designing effective strategies that reduce the risk of cataract formation.

## Figures and Tables

**Table 1:** Summary of animal studies and the effects of dietary vitamin C supplementation or depletion of vitamin C on the lens.

	Species	Method of Cataract Induction	Type of Cataract	Vitamin C Elevation or Depletion	Parameters Measured	Outcome	Ref
**In vitro studies**	Rats Wistar/NIN inbred strain (3 months old)	Irradiation of lenses at 300 nm for 24 h	No lens opacification	Lenses irradiated in media containing 2 mM ascorbic acid or 2 μM α-tocopherol acetate or 10 μm β-carotene	-Enzyme activity of glycolysis pathways (hexokinase, glucose-6-phosphate dehydrogenase, aldose reductase) -Na, K- ATPase activity -Lipid peroxidation	Addition of ascorbic acid or α-tocopherol or β-carotene to the media, reduced lipid peroxidation and increased activities of enzyme involved in the glycolysis hexomonophosphate pathway	[86]
	Rabbit lens epithelial cells	Buthionine sulfoximine	Not reported. Lenses exhibited a depletion of ~75% GSH	Cells were cultured in 25–50 µM vitamin C or 5–40 µM vitamin E at the same time as BSO treatment for 24 h and then exposed to H_2_O_2_ for 1 h	-Cell viability: MTS assay, LDH assay -GSH/GSSG levels	Supplementation of vitamin C and vitamin E protects GSH-depleted lens epithelial cells by reducing levels of GSSG	[87]
	Mice CD-1 (25g)	Lenses were cultured in xanthine, xanthine oxidase, and uricase	Not stated	Lenses were cultured in 2 mM ascorbate and ROS-inducing reagents along with ^86^RbCl	-Membrane transport activity -ATP levels -GSH levels	ROS agents decreased membrane transport activity, ATP and GSH. Ascorbate minimized these effects significantly	[88]
	Calf lenses	NA	NA	Water-insoluble proteins from aged normal human lenses, early stage brunescent cataract lenses and calf lens proteins were reacted with or without 20 mM ascorbate in air for 4 weeks	-Protein modifications (glycation reactions)	AGEs present in aged and cataractous human lenses eluted at the same retention times as those from ascorbic acid glycated calf lens proteins, suggesting that the yellow chromophores in brunescent lenses represent AGEs due to ascorbic acid glycation	[37]
				Water-insoluble proteins from aged normal human lenses, early stage brunescent cataract lenses and calf lens proteins were reacted with or without 20 mM ascorbate in air for 4 weeks	-Amino acid modifications -Protein modifications (glycation reactions)	LC-MS revealed that the majority of the major modified amino acids present in early stage brunescent cataract lens proteins were as a result of ascorbic acid modification	[89]
				Incubation of calf lens extracts with either 10 mM ascorbic acid, 20 mM sorbitol, or 20 mM glucose for 8 weeks	-Protein precipitation and browning -Cross linking of proteins -Protein modifications (glycation)	Only ascorbic acid induced the formation of high molecular weight aggregates with extensive browning	[90]
	Bovine lens crystallin proteins	NA	NA	Bovine lens crystallin proteins incubated with [^14^C] ascorbic acid for 1 month and the fluorescence spectrum compared to human cataractous lenses	-Browning -Binding of Ascorbic Acid Oxidation Products to Proteins. -Comparison of fluorescence Spectra	Formation of brown condensation products correlated with increased protein radioactivity. Fluorescence spectrum of condensation products was similar to spectrum of human cataractous lenses	[91]
				Bovine lens β-crystallin incubated with increasing concentrations of sugars and sugar derivatives for a period of 2 weeks in the dark at 37 °C	-Protein precipitation and browning -Cross linking of proteins	Protein precipitation and browning reaction was observed with both vitamin C and DHA. No reaction was seen with several other sugars suggesting that vitamin C is a significant glycating agent	[92]
**In vivo studies**	Guinea pigs (between 280 and 320 g)	UV-B (0.25–0.75 J/cm^2^) 10 min exposure time	Not mentioned	Vitamin C depletion via guinea pigs fed an ascorbate-deficient diet	-DNA damage (DNA single strand breaks)	Lenses from ascorbate deficient guinea pigs showed 50% more DNA damage than those from normal guinea pigs after UV exposure	[93]
	Rats Harlan Sprague-Dawley (300 g)	UV-B (0.25–0.75 J/cm^2^) 10 min exposure time	Not mentioned	IP injections of sodium ascorbate (1 g/kg)	-DNA damage (DNA single strand breaks)	Increase in vitamin C in AH and lenses; 50% decrease in UV-induced DNA strand breaks compared to non-ascorbate injected rats	[93]
	Guinea pigs (56 days old, 500–600 gm each)	NA	NA	High dietary ascorbate (50 mg/day) vs. low dietary ascorbate (2 mg/day) for 21 weeks. Lens homogenates exposed to UV light.	-Protein damage (high-molecular-weight aggregates and enhanced loss of exopeptidase activity)	Markers of light-induced protein damage were reduced in the HDA animals compared to LDA animals	[94]
	Rat Sprague-Dawley (p8-p21)	IP admin of sodium selenite at postnatal day 10	Nuclear	Daily IP dose of sodium ascorbate (0.3 mmol) at postnatal day 8 until postnatal day 25	-ATP -GSH -MDA -Soluble protein -Lens transparency	Ascorbate was able to restore ATP and GSH levels and reduced MDA levels that were altered in sodium selenite lenses. Significantly reduced cataracts in animals administered with ascorbate	[95]
	Senescence marker protein-30 knockout (KO) mice	UVR-B (200 mW/cm^2^) for 100 s twice a week for 3 weeks	Anterior subcapsular cataract	Fed a vitamin C sufficient diet (1.5 g/L) or vitamin C deficient diet (0.0375 g/L) and then exposed to UV-B	-Lens morphology -Protein content -Lens transparency	Less extensive opacities	[96]
	Rats Wistar (18–20 months)	Streptozotocin	Cortical	STZ diabetic rats were fed a Vitamin C (1 g ascorbate/kg feed) and vitamin E (600 mg dl-α-tocopherol acetate/kg feed) supplemented diet	-Lipid peroxidation -GSH -GSH-Px activity	Lowered lipid peroxidation levels in the lens Increased GSH-Px activity No mention of effects on lens opacities	[97]
	Rats Wistar (age not specified)	Streptozotocin	Cortical	STZ diabetic rats were fed vitamin C at 0%, 0.3%, and 1.0% (w/w) to rodent chow	-Membrane integrity -ATP -Lens transparency	Treatment of diabetic group with vit C at 0.3% and 1% lead to decrease in leakage of γ-crystallins into the aqueous and vitreous humor. A reduction in cataract was detected for the 1% dietary vitamin C group	[98]
	Rats Wistar (12 weeks)	Streptozotocin	Cortical	IP administered with vitamin E (20 mg over 24 h), selenium (0.3 mg over 24 h), vitamin E (20 mg) and selenium combination (0.3 mg over 24 h), or vitamin C (30 mg over 24 h). On the fourth day after injection, IP injections of STZ were administered.	-MDA -GSH -GPx activity	Vitamins C and E and selenium can protect the lens against oxidative damage, but the effect of vitamin C appears to be much greater than that of vitamin E and selenium. No mention of lens opacities	[99]
	Transgenic mouse in which SVCT2 is overexpressed	NA	At 12 months of age, transgenic lenses were a yellow colour similar to that observed in older human lenses	Transgenic lenses contained 10-fold greater vitamin C and 25-fold more DHA than WT lenses	-Protein modifications	Transgenic lenses contained increased levels of vitamin C derived advanced ascorbylation end products which are also known to be present in the aging human lens	[100]
	Guinea pigs (6–9 weeks)	UVR-B (80 kJ/m^2^)	Superficial anterior cataract	Drinking water supplemented with or without 5.5 mm l-ascorbate for 4 weeks. After supplementation, animals were exposed in vivo to 80 kJ/m^2^ UVR-B.	-Lens transparency via forward light scattering measurements	Cataract develops in lenses exposed to UVR-B both in animals given drinking water that is supplemented with ascorbate and those whose drinking	[101]

**Table 2:** Human studies investigating the effect of vitamin C supplements on the development of cataracts.

Study, Type	Nutrients	Population	Disease Outcome	Results	Year, Author
Age-related cataract in a randomized trial of vitamins E and C in men. Eight years of treatment and follow-up RCT	Vitamin E 400 IU or placebo on alternate days and vitamin C 500 mg of or placebo daily	Participants: 11,545 United States male ≥50 years	Incidence of age-related cataract	No significant beneficial or harmful effect on the risk of cataract. HR 1.02; 95% confidence interval, 0.91–1.14	[116]
The Swedish mammography cohort study follow up. 8.2 years of follow-up Population-based, prospective cohort of women.	Vitamin C (approximately 1 g) Vitamin c within a multivitamin supplement (approximately 60 mg)	Participants: 24,593 Sweden female 49–83 years	Incidence of age-related cataracts	The use of vitamin C supplements may be associated with a higher risk of age-related cataract among women. The multivariable HR for vitamin C supplement vs. nonusers was 1.25 (95% CI: 1.05, 1.50). The HR for the duration of 10 y of use before baseline was 1.46 (95% CI: 0.93, 2.31). The HR for the use of multivitamins containing vitamin C was 1.09 (95% CI: 0.94, 1.25).	[117]
High-dose Supplements of Vitamins C and E, Low-Dose Multivitamins, and the Risk of Age-Related Cataract Follow-up of 8.4 years Cohort	Vitamin C and vitamin E as single supplements was estimated to be 1 g and 100 mg, respectively. Multivitamins were estimated to contain 60 mg of vitamin C and 9 mg of vitamin E	Participants: 31,120 Sweden male 45–79 years	Risk of age-related cataract	Use of high-dose (but not low-dose) single vitamin C supplements increased the risk of age-related cataract. The multivariable- adjusted HR for men using vitamin C supplements only was 1.21 (95% confidence interval (CI): 1.04, 1.41) in a comparison with that of non-supplement users. The HR for long-term vitamin C users (≥10 years before baseline) was 1.36 (95% CI: 1.02, 1.81). The risk of cataract with vitamin C use was stronger among older men (>65 years) (HR = 1.92, 95% CI: 1.41, 2.60) and corticosteroid users (HR = 2.11, 95% CI: 1.48, 3.02)	[118]

Abbreviations: RCT, Randomized Control Trials, HR, Hazard Ratio, OR, Odd Ration, HRT, Hormone Replacement Therapy.

**Table 3:** Human studies investigating the effect of diets high in vitamin C on the development of cataracts.

Study, Design	Nutrients Studied	Population	Disease Outcome	Results	Ref
The India Study of Age-related Eye Disease (INDEYE study) a population-based study. Cross-sectional analytic study	Vitamin C and inclusion of other antioxidants (lutein, zeaxanthin, retinol, β-carotene, and α-tocopherol)	Participants:5638 North and South India Male and female ≥60 years	Incidence of cataract in the Indian setting	Vitamin C was inversely associated with cataract (adjusted OR for highest to lowest quartile = 0.61; 95% confidence interval (CI), 0.51–0.74; *p* = 1.1 × 10^−6^). Similar results were seen by type of cataract: nuclear cataract (adjusted OR 0.66; CI, 0.54–0.80; *p* = 0.0001), cortical cataract (adjusted OR 0.70; CI, 0.54–0.90; *p* = 0.002), and PSC (adjusted OR 0.58; CI, 0.45–0.74; *p* = 0.00003)	[128]
Healthy Diets and the Subsequent Prevalence of Nuclear Cataract in Women. Participated in the Carotenoids in Age-Related Eye Disease Study—7 years follow up	Vitamin C (40 vs. 207 mg/d); vitamin E (3 vs. 11 mg/d)	Participants: 1808 United States female 50–79 years	Prevalence of nuclear cataract in women.	Adjustment of the OR for nuclear cataract among women with high vs. low HEI-95 scores, for vitamin C intake from foods attenuated the ORs (Multivariate OR (95%CI) = 0.76 (0.50–1.15), suggesting that higher vitamin C intakes partly explained the associations with HEI-95 dietary assessment. There was a significant linear trend for a protective association of vitamin C intake from foods	[132]
The European Eye Study (EUREYE study). Recruited during 1-year period. Multi-center cross-sectional population-based study	Carotenoids, vitamins C (107 mg/d) and E	Participants: 599 Spain Male/female ≥65 years	Prevalence of cataract with fruit and vegetable intake	High daily intakes of fruit and vegetables and vitamin c were associated with a significantly decreased prevalence of cataract or cataract surgery (*p* for trend = 0.008). Increasing quartiles of dietary intakes from 107 mg/d of vitamin C showed a significant decreasing association with prevalence of cataract or cataract extraction (*p* for trend = 0.047)	[129]
Diet and cataract. Case-control study	Carbohydrates carotene vitamins C and E	Participants: 314 cataract cases and 314 controls Greece Male/Female 45–85 years	Association between diet and risk of cataract in Athens	There was a protective association between cataract risk and intake of vitamin c (OR = 0.50, *p* \ 0.001 for cataract overall; OR = 0.55, *p* \ 0.001 for nuclear cataract; OR = 0.30, *p*\0.001 for PSC)	[131]

Abbreviations: EUREYE, The European Eye Study, HR, Hazard Ratio, OR, Odd Ratio.
